# Gestational age and 1-year hospital admission or mortality: a nation-wide population-based study

**DOI:** 10.1186/s12887-017-0787-y

**Published:** 2017-01-18

**Authors:** Silvia Iacobelli, Evelyne Combier, Adrien Roussot, Jonathan Cottenet, Jean-Bernard Gouyon, Catherine Quantin

**Affiliations:** 1 Centre d’Etudes Périnatales de l’Océan Indien, CHU Sud Réunion, La Réunion, France; 2 Réanimation Néonatale et Pédiatrique, Néonatologie, CHU La Réunion, France; 3 Centre d’Epidémiologie des Populations, Université de Bourgogne, EA4184 Dijon, France; 4 Service de Biostatistique et d’Informatique Médicale (DIM), CHRU Dijon, Dijon, F-21000 France; 5 INSERM, CIC 1432, Dijon, France; 6 Clinical Investigation Center, Clinical epidemiology/Clinical trials unit, Dijon University Hospital, Dijon, France; 7 Biostatistics, Biomathematics, Pharmacoepidemiology and Infectious Diseases (B2PHI), INSERM, UVSQ, Institut Pasteur, Université Paris-Saclay, Villejuif, France

**Keywords:** Moderate preterm, Late preterm, Early Term, Morbidity, Mortality, Hospital discharge data, PMSI

## Abstract

**Background:**

Describe the 1-year hospitalization and in-hospital mortality rates, in infants born after 31 weeks of gestational age (GA).

**Methods:**

This nation-wide population-based study used the French medico-administrative database to assess the following outcomes in singleton live-born infants (32–43 weeks) without congenital anomalies (year 2011): neonatal hospitalization (day of life 1 – 28), post-neonatal hospitalization (day of life 29 – 365), and 1-year in-hospital mortality rates. Marginal models and negative binomial regressions were used.

**Results:**

The study included 696,698 live-born babies. The neonatal hospitalization rate was 9.8%. Up to 40 weeks, the lower the GA, the higher the hospitalization rate and the greater the likelihood of requiring the highest level of neonatal care (both *p* < 0.001). The relative risk adjusted for sex and pregnancy-related diseases (aRR) reached 21.1 (95% confidence interval [CI]: 19.2-23.3) at 32 weeks. The post-neonatal hospitalization rate was 12.1%. The raw rates for post-neonatal hospitalization fell significantly from 32 – 40 and increased at 43 weeks and this persisted after adjustment (aRR = 3.6 [95% CI: 3.3–3.9] at 32 and 1.5 [95% CI: 1.1–1.9] at 43 compared to 40 weeks). The main causes of post-neonatal hospitalization were bronchiolitis (17.2%), gastroenteritis (10.4%) ENT diseases (5.4%) and accidents (6.2%). The in-hospital mortality rate was 0.85‰, with a significant decrease (*p* < 0.001) according to GA at birth (aRR = 3.8 [95% CI: 2.4–5.8] at 32 and 6.6 [95% CI: 2.1–20.9] at 43, compared to 40 weeks.

**Conclusion:**

There’s a continuous change in outcome in hospitalized infants born above 31 weeks. Birth at 40 weeks gestation is associated with the lowest 1-year morbidity and mortality.

## Background

The increased vulnerability of moderate preterm infants (moderate PT) (32^0/7^-33^6/7^ weeks of gestation) and late preterm infants (late PT) (34^0/7^–36^6/7^) in the neonatal period is now widely accepted [1–5], and undoubtedly these infants have both excess morbidity and mortality [1–9]. Recently, it has been shown that this is also relevant for early term infants (early PT) (37^0/7^–38^6/7^) compared with infants born full term (39^0/7^–40^6/7^) and post-date (≥41^0/7^) [1, 2, 4, 6–10].

Moreover, studies have identified an increased risk of hospitalization for late PT compared with full term infants within the year after discharge [11–15]. A large epidemiological study conducted in California showed that the likelihood of any hospitalization in the first year of life decreased with advancing gestational age (GA), even inside the subgroup of infants categorized as late PT [11].

Therefore, the hypothesis of a continuous change in babies’ outcome according to completed weeks of gestation should be evaluated before revisiting the usual categorization of GAs. More information is also needed to: i) identify the respective contribution of each week of gestation in the prognosis of infants born after 31 weeks of gestation; ii) understand the associated morbidities, in order to reduce the hospitalization rate of these babies, as they account for a large proportion of births [11].

The objective of this nation-wide population-based study was to evaluate, in infants born after 31 weeks of gestation, the hospitalization rate and in-hospital mortality rate in the first year after birth, according to GA at birth.

## Methods

### Data sources

The data used for the study were recorded for births in 2011 and for hospitalizations in 2011–2012, in the French medico-administrative database PMSI (Programme de Médicalisation des Système d’Information), [16] gathering discharge abstracts from all hospitals in France. Diagnoses and procedures are coded according to the International Classification of Diseases (ICD-10) [17] and to the French Classification of Medical Procedures (CCMP) [18]. Quality control procedures are carried out a posteriori on samples by medical inspectors.

GA recording on the PMSI became mandatory in 2009 [19]. In France 99.6% of births occur in hospital and thus are recorded in the PMSI. [20].

The main perinatal variables necessary for this study were recently found to be robust when the PMSI (2010) was compared with national vital statistics and to a national perinatal survey [19]. Moreover, this database allows the linkage of discharge abstracts of successive hospitalizations for the same infant [16].

### Study population

The study concerned the year 2011 and all public and private hospitals of Metropolitan France. Live-born singletons with GA between 32 and 43 weeks were eligible.

Exclusion criteria were births outside Metropolitan France, stillbirths (defined as both antepartum and intrapartum fetal deaths after 22 weeks of gestation), multiple births, births with congenital malformations or chromosomal abnormalities, and births when the discharge abstract did not contain the anonymized identifier needed to link hospitalizations for the same infant.

### Variables

Outcomes of interest were neonatal hospitalization, post-neonatal hospitalization and in-hospital death up to the first year of life.

Neonatal hospitalization was defined as any hospital stay in the first 28 days of life, other than the postpartum stay in maternity yard (well born nursery), whatever the duration of the hospitalization. Day 1 of life was defined as the day of birth. This day 1 – 28 period (d1-day) could include hospital admissions following the birth or ensuing admissions in any of the following neonatal units: level IIA (special care nursery), level IIB (intermediate neonatal care) or level III (intensive neonatal care – NICU), or in pediatric or surgical yards.

Post-neonatal hospitalization was defined as any hospital stay with admission between day 29 and day 365 of life (d29-d365) in neonatal units, or in pediatric or surgical yards.

The total duration of the hospital stay was calculated, as the sum of the stays mentioned on the successive discharge abstract. The discharge abstracts for post-neonatal hospitalizations were gathered together according to the main coded diagnoses. Deaths were identified from the discharge modality reported in the discharge abstracts. Age at the time of death was calculated by adding the length of hospital stay to the age in days at admission.

Rates of hospitalization and in-hospital death were considered according to GA.

GA was estimated as the duration of gestation at the time of delivery in completed weeks. GA was calculated according to the early antenatal ultrasound scan and by the date of the last menstrual period. In France, 90% of pregnant women have an early ultrasound to determine the date of conception [19].

### Statistical analysis

We analyzed the impact of GA on infant mortality, morbidity and its different components, successive hospitalizations (indication of admission, level of care, duration of hospitalization) occurring during the first year of life. In this study, 40 weeks of gestation was chosen as the reference GA, because this was associated with the lowest rate of neonatal hospitalization.

For bivariate analyses of qualitative variables, we used the Chi Square test to compare percentages and the Somers’ D to analyze trends. Mean values of quantitative variables were compared using linear regressions.

In order to analyze the impact of GA alone, for each variable of interest in the management of the pregnancy, the delivery and the post-partum period that could have affected the state of health of the newborns in each establishment, we used marginal models and negative binomial regressions, which make it possible to take into account the hierarchical structure of the data and their overdispersion [21]. We adjusted for sex, pregnancy-related diseases (coded as P01 “Fetus and newborn affected by maternal complications of pregnancy” and P02 “Fetus and newborn affected by complications of placenta, cord and membranes) and neonatal hospitalization when required.

FREQ, REG and GENMOD procedures of SAS® version 9.3 (SAS Institute Inc., Cary, NC, USA) were used for the analyses.

This study was approved by the National Committee for data protection (Commission Nationale de l'Informatique et des Libertés, registration number 1576793) to access and use the national PMSI database and was conducted in accordance with French legislation. Written consent was not needed for this study.

## Results

### Population

First, 818 595 discharge abstracts were identified and corresponded to all births recorded in the national PMSI for the year 2011. The study selected 696 698 live singleton births (85.1% of all births) with GA between 32 and 43 weeks and without exclusion criteria. There were 696 358 infants still alive at day 29 of life.

Table 1 shows the distribution of births by GA among boys and girls. Overall, the sex ratio (number of live-born boys/number of live-born girl) was 1.03 with a U-shaped distribution around 40 weeks. Up to 40 weeks of gestation, the shorter the term, the higher the sex ratio. Infants born at 43 weeks of gestation represented only 0.03% of the entire population.

**Table 1:** Distribution by gestational age (GA) and sex among 696 698 singleton live-born babies ≥ 32 weeks of gestation (France, 2011)

GA	Live-born babies	Live-born boys	Live-born girls	Sex-ratio^a^
	Number (%)	Number (%)	Number (%)	
32	1741 (0.3)	961 (0.3)	780 (0.2)	1.23
33	2529 (0.4)	1400 (0.4)	1129 (0.3)	1.24
34	4552 (0.7)	2437 (0.7)	2115 (0.6)	1.15
35	7962 (1.1)	4322 (1.2)	3640 (1.1)	1.19
36	16 072 (2.3)	8659 (2.5)	7413 (2.2)	1.17
37	39 585 (5.7)	20 954 (5.9)	18 631 (5.4)	1.12
38	105 292 (15.1)	54 367 (15.4)	50 925 (14.8)	1.07
39	192 426 (27.6)	97 078 (27.5)	95 348 (27.7)	1.02
40	194 682 (27.9)	96 314 (27.3)	98 368 (28.6)	0.98
41	125 942 (18.1)	63 175 (17.9)	62 767 (18.3)	1.01
42	5688 (0.8)	2980 (0.8)	2708 (0.8)	1.10
43	227 (0.03)	129 (0.04)	98 (0.03)	1.32
TOTAL	696 698 (100%)	352 776 (50.6%)	343 922 (49.4%)	1.03

*p* (Somer’s D) < 0.001: significant trend for the sex-ratio according to gestational age

*p* (Chi square) test < 0.001: significant difference for the distribution of sex according to gestational age

^a^sex-ratio = number of live-born boys/number of live-born girls

### Neonatal hospitalization (d1-d28)

Of the total population of infants, 68 067 (9.8%) were admitted to hospital between d1 and d28. Of these, 56 016 (82.3%) were admitted to a neonatal unit and 12 051 to a pediatric or surgical ward. Table 2 shows the hospitalization rate by GA and sex among the study population. Up to 40 weeks, the lower the gestational age, the higher the hospitalization rate. Boys were consistently more likely than girls to be hospitalized (10.5% vs 9.0%). The sex ratio of hospitalized babies was 1.20 and it did not vary significantly with GA.

**Table 2:** Neonatal hospitalization rate by gestational age (GA) and sex among 696 698 singleton live-born babies ≥ 32 weeks of gestation (France, 2011)

GA	All babies	Boys	Girls	Sex-ratio^a^
	Number (%)	Number (%)	Number (%)	
32	1638 (94.1)	910 (94.7)	728 (93.3)	1.25
33	2394 (94.7)	1328 (94.9)	1066 (94.4)	1.25
34	4146 (91.1)	2225 (91.3)	1921 (90.8)	1.16
35	5695 (71.5)	3105 (71.8)	2590 (71.2)	1.20
36	5890 (36.7)	3248 (37.5)	2642 (35.6)	1.23
37	5925 (15.0)	3242 (15.5)	2683 (14.4)	1.21
38	9208 (8.8)	4926 (9.1)	4282 (8.4)	1.15
39	12 358 (6.4)	6859 (7.1)	5499 (5.8)	1.25
40	11 876 (6.1)	6437 (6.7)	5439 (5.5)	1.18
41	8486 (6.7)	4636 (7.3)	3850 (6.1)	1.20
42	431 (7.6)	236 (7.9)	195 (7.2)	1.21
43	20 (8.8)	13 (10.1)	7 (7.1)	1.86
Total	68 067 (9.8)	37 165 (10.5)	30 902 (9.0)	1.20

*p* (Chi Square) = 0.2461: no significant difference for the distribution of sex according to gestational age

*p* (Somer’s D) < 0.0001: significant trend for the sex-ratio according to gestational age

^a^sex-ratio = number of hospitalized boys/number of hospitalized girls

Up to 40 weeks, the lower the GA, the greater the likelihood of requiring the highest level of neonatal care. The same statistically significant trend was found for infants whose highest level of care was special care nursery or intermediate neonatal care (*p* < 0.001) (Table 3).

**Table 3:** Level of neonatal care required among 696 698 singleton live-born babies with gestational age (GA) ≥ 32 weeks of gestation during neonatal hospitalization (France, 2011)

GA	No admission to a neonatal care unit	Special care nursery	Intermediate neonatal care unit	NICU
	Number (%)	Number (%)	Number (%)	Number (%)
32	109 (6.3)	204 (11.7)	814 (46.75)	614 (35.3)
33	141 (5.6)	723 (28.6)	1164 (46.03)	501 (19.8)
34	420 (9.2)	2096 (46.1)	1492 (32.78)	544 (12.0)
35	2333 (29.3)	3766 (47.3)	1405 (17.65)	458 (5.8)
36	10 491 (65.3)	4104 (25.5)	1058 (6.58)	419 (2.6)
37	34 552 (87.3)	3697 (9.3)	947 (2.39)	389 (1.0)
38	98 188 (93.3)	5338 (5.1)	1299 (1.23)	467 (0.4)
39	183 445 (95.3)	6753 (3.5)	1663 (0.86)	565 (0.3)
40	186 030 (95.6)	6304 (3.2)	1730 (0.89)	618 (0.3)
41	119 413 (94.8)	4795 (3.8)	1285 (1.02)	449 (0.4)
42	5352 (94.1)	222 (3.9)	86 (1.51)	28 (0.5)
43	208 (91.6)	13 (5.7)	2 (0.88)	4 (1.8)
Total	640 682 (92.0)	38 015 (5.5)	12 945 (1.9)	5056 (0.7)

*p* (Somer’s D) < 0.001 significant trend for the level of neonatal care according to gestational age

After adjustment for sex and pregnancy-related diseases, the adjusted relative risk (aRR) for hospital admission between d1 and d28 was inversely related to GA and reached 21.1 (95% confidence interval [CI]: 19.2–23.3) at 32 weeks of gestation (Table 4).

**Table 4:** Neonatal admission (d1-d28) to any neonatal care unit, according to gestational age (GA) among 696 698 singleton live-born babies (France, 2011) (*N* = 56 016–8.0%)

GA	Hospitalized babies	RR [95% CI]^a^	aRR [95% CI]^b^
	Number (%)		
32	1632 (93.7)	20.6 [18.8–22.5]	21.1 [19.2–23.2]
33	2388 (94.4)	20.8 [19.0–22.8]	21.2 [19.4–23.2]
34	4132 (90.8)	19.3 [17.7–21.0]	19.7 [18.1–21.5]
35	5629 (70.7)	14.4 [13.3–15.6]	14.7 [13.6–15.9]
36	5581 (34.7)	7.1 [6.6–7.6]	7.2 [6.7–7.7]
37	5033 (12.7)	2.7 [2.5–2.8]	2.7 [2.6–2.9]
38	7104 (6.7)	1.5 [1.4–1.6]	1.5 [1.4–1.6]
39	8981 (4.7)	1.0 [1.0–1.1]	1.0 [1.0–1.1]
40	8652 (4.4)	–	–
41	6529 (5.2)	1.2 [1.1–1.2]	1.2 [1.1–1.2]
42	336 (5.9)	1.3 [1.2–1.5]	1.4 [1.2–1.6]
43	19 (8.4)	1.5 [0.7–3.5]	1.6 [0.7–3.6]
Total	56 016 (8.0)		

*p* (Somer’s D) < 0.001: significant trend for neonatal admission according to gestational age

^a^RR: relative risk

^b^aRR: relative risk adjusted for sex and pregnancy-related diseases (ICD10 codes P01 and P02)

A similar and significant trend was observed for admission to a NICU at 32 weeks (aRR = 98.3 [95% CI: 82.9–116.7]) (Table 5).

**Table 5:** Neonatal admission (d1-d28) to NICU, according to gestational age (GA) among 696 698 singleton live-born babies (France, 2011) (*N* = 5056 – 0.7%)

GA	Hospitalized babies	RR [95% CI]^a^	aRR [95% CI]^b^
	Number (%)		
32	614 (35.3)	100.1 [84.4–118.8]	98.3 [82.9–116.7]
33	501 (19.8)	58.5 [49.3–69.4]	57.0 [48.0–67.7]
34	544 (12.0)	33.3 [28–39.5]	32.6 [27.5–38.6]
35	458 (5.8)	17.1 [14.5–20.1]	17.0 [14.5–20]
36	419 (2.6)	8.1 [6.9–9.5]	8.1 [6.9–9.5]
37	389 (1.0)	3.0 [2.6–3.4]	3.0 [2.6–3.5]
38	467 (0.4)	1.4 [1.2–1.6]	1.4 [1.2–1.6]
39	565 (0.3)	0.9 [0.8–1.0]	0.9 [0.8–1.0]
40	618 (0.3)	–	–
41	449 (0.4)	1.1 [1.0–1.3]	1.1 [1.0–1.3]
42	28 (0.5)	1.7 [1.2–2.4]	1.7 [1.2–2.4]
43	4 (1.8)	6.6 [2.7–16.2]	6.9 [2.8–16.9]
Total	5056 (0.7)		

*p* (Somer’s D) < 0.001: significant trend for NCIU admission according to gestational age

^a^RR: relative risk

^b^aRR: relative risk adjusted for sex and pregnancy-related diseases (ICD10 codes P01 and P02)

### Post-neonatal hospitalization (d29-d365)

Among the 696 358 infants still alive at d29, 84 301 were admitted to hospital in the period d29–d365 (12.1%) for a total of 120 482 hospital stays. Among these infants, 15 261 (18.1%) had already been hospitalized in the neonatal period and the rate of post-neonatal hospitalizations was higher in infants who had already been hospitalized in the neonatal period (22.5 vs 11.0%; *p* < 0.001). Neonatal hospitalization following birth increased the risk of post-neonatal hospitalization in the first year of life by 1.8 (95% CI: 1.8 – 1.9) after adjustment for sex and pregnancy-related diseases.

The raw rates for post-neonatal hospitalization according to GA fell steadily from 32 to 40 and increased at 43 weeks of gestation. The trend was significant (*p* < 0.001) and persisted after adjustment for sex, pregnancy-related disease and neonatal hospitalization. The aRR of post-neonatal hospitalization was 3.6 (95% CI: 3.3 – 3.9) at 32 and 1.5 (95% CI: 1.1 – 1.9) at 43 compared to 40 weeks of gestation (RR = 1) (results shown in Table 6).

**Table 6:** Post-neonatal admission (d29-d265) according to gestational age (GA) among 696 358 singleton babies still alive at d29 (France, 2011) (*N* = 84 301, 12.1%)

GA	Hospitalized babies	RR [95% CI]^a^	aRR [95% CI]^b^	aRR_1_ [95% CI]^c^
	Number (%)			
32	727 (42.2)	3.6 [3.3–3.9]	3.6 [3.3–3.9]	2.2 [2.1–2.4]
33	781 (31.1)	2.7 [2.5–2.8]	2.6 [2.5–2.8]	1.6 [1.5–1.8]
34	1193 (26.3)	2.2 [2.1–2.4]	2.2 [2.1–2.4]	1.4 [1.3–1.5]
35	1706 (21.5)	1.8 [1.7–1.9]	1.8 [1.7–1.9]	1.3 [1.2–1.3]
36	2775 (17.3)	1.5 [1.4–1,5]	1.5 [1.4–1.5]	1.3 [1.2–1.3]
37	5840 (14.8)	1.3 [1.3–1.3]	1.3 [1.3–1.3]	1.2 [1.2–1.3]
38	13 544 (12.9)	1.2 [1.1–1.2]	1.2 [1.1–1.2]	1.1 [1.1–1.2]
39	22 599 (11.8)	1.1 [1.0–1.1]	1.1 [1.0–1.1]	1.1 [1.0–1.1]
40	21 326 (11.0)	–	–	–
41	13 187 (10.5)	0.9 [0.9–1.0]	0.9 [0.9–1.0]	0.9 [0.9–1.0]
42	584 (10.3)	0.9 [0.8–1.0]	0.9 [0.8–1.0]	0.9 [0.8–1.0]
43	39 (17.3)	1.5 [1.1–1.9]	1.5 [1.1–1.9]	1.5 [1.1–2.0]
Total	84 301 (12.1)			

*p* (Somer’s D) < 0.001: significant trend for post-neonatal admission according to gestational age

^a^RR: relative risk

^b^aRR: relative risk adjusted for sex, pregnancy-related diseases (ICD10 codes P01 and P02)

^c^aRR_1_: relative risk adjusted for sex, pregnancy-related diseases (ICD10 codes P01 and P02) and hospitalization in the first 28 days of life

Infections (Table 7) were the main reason for post-neonatal hospitalization (40.2%) and these were principally bronchiolitis (17.2%), gastroenteritis (10.4%) and ear/nose/throat infections (5.4%). In 6.1% of cases, hospital admission was due to accidents and 8.7% of hospital stays were related to surgery. Taken individually, each of the other causes accounted for less than 2% of hospital stays.

**Table 7:** Causes of hospital admissions between d29 and d365 in 696 358 singleton infants alive at d29 and born ≥ 32 weeks of gestation (France, 2011)

Main diagnosis	Hospital stays	Infants
	Number (%)^a^	Number (%)^b^
Total	120 482 (17.3)	84 301 (12.1)
Infections	48 187 (40.0)	41 231 (5.9)
Including:		
Bronchiolitis	20 698 (17.2)	18 370 (2.6)
Gastroenteritis	12 526 (10.4)	11 740 (1.7)
Ear, nose, throat	6522 (5.4)	6192 (0.9)
Accidental	7323 (6.1)	6815 (1.0)
Including:		
Trauma	5972 (5.0)	5719 (0.8)
Burns	452 (0.4)	272 (0.04)
Intoxications	206 (0.2)	203 (0.03)
Inhalation of foreign bodies	232 (0.2)	219 (0.03)
Abuse	91 (0.1)	86 (0.01)
Cancer	791 (0.7)	122 (0.02)
Sub total	56 392 (46.8)	48 254 (6.9)
Surgical	10 530 (8.7)	9655 (1.4)
Others	64 090 (53.2)	36 047 (5.2)

^a^compared with the total number of Discharges Abstracts (*N* = 120 482)

^b^compared with the total number of infants alive at d28 (*N* = 696 358)

When looking specifically at hospitalizations in neonatal units, these accounted for a total of 490 986 days in the study population.

Moderate, late, early PT, full-term and post-date infants accounted respectively for 22.5, 35.9, 15.6, 25.6 and 0.4% of these hospitalizations.

**Fig. 1:**
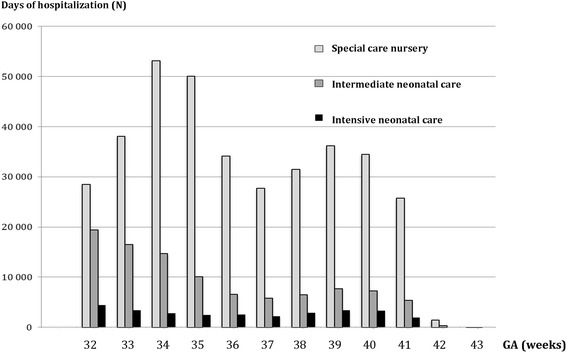
Cumulative days of hospitalization among 696 698 singleton live-born babies with GA ≥ 32 weeks of gestation (France, 2011)

The total number of hospital days according to the neonatal unit level of care and to GA at birth is shown in Fig. 1.

### In-hospital mortality in the first year of life

There were 598 in-hospital deaths (in-hospital mortality rate: 0.85/1000), of which 340 occurred from d1 to d28 (0.48/1000) and 258 from d29 to d365 (0.48/1000). For the two periods (Table 8), there was a significant decrease (*p* < 0.001) in raw mortality rates according to GA. Both unadjusted and adjusted RR (for sex, pregnancy-related disease and neonatal hospitalization) for death was significantly increased below 38 and at 43 weeks of gestation, the reference always being 40 weeks of gestation (Table 8).

**Table 8:** Hospital mortality rate in the first year (d1-d365), in the neonatal (d1-d28) and the post-neonatal (d29-d365) periods, according to gestational age (GA) among 696 698 singleton live-born babies with GA ≥ 32 weeks of gestation (France, 2011)

	**GA**	Deaths	RR [95% CI]^a^	aRR [95% CI]^b^	aRR_1_ [95% CI]^c^
		Number (‰)			
First year mortality	**32**	25 (1.44)	21.7 [14.2–33.3]	21.9 [14.3–33.4]	3.8 [2.4–5.8]
	**33**	34 (1.34)	19.9 [13.0–30.5]	19.9 [13.0–30.5]	3.4 [2.2–5.5]
	**34**	31 (0.68)	10.2 [6.7–15.4]	10.3 [6.8–15.5]	1.8 [1.2–2.8]
	**35**	35 (0.44)	6.4 [4.4–9.3]	6.5 [4.4–9.5]	1.5 [1.0–2.2]
	**36**	43 (0.27)	4.1 [2.9–5.8]	4.2 [3.0–5.9]	1.6 [1.1–2.3]
	**37**	64 (0.16)	2.5 [1.8–3.5]	2.6 [1.9–3.5]	1.8 [1.3–2.5]
	**38**	80 (0.08)	1.3 [1.0–1.8]	1.4 [1.0–1.8]	1.2 [0.9–1.6]
	**39**	108 (0.06)	1.0 [0.8–1.4]	1.0 [0.8–1.4]	1.0 [0.8–1.3]
	**40**	107 (0.05)	–	–	–
	**41**	66 (0.05)	0.9 [0.7–1.2]	0.9 [0.7–1.2]	0.9 [0.7–1.2]
	**42**	4 (0.07)	1.2 [0.4–3.2]	1.2 [0.4–3.2]	1.2 [0.4–3.2]
	**43**	1 (0.44)	6.9 [1.4–33.3]	7.0 [1.4–34.1]	6.6 [2.1–20.9]
	**Total**	**598 (0.86)**			
Neonatal mortality	**32**	19 (1.09)	29.1 [16.9–50.0]	29.5 [17.3-50.5]	5.1 [2.9–8.9]
	**33**	19 (0.75)	20.9 [12.0–36.4]	21.1 [12.1–36.8]	3.5 [1.9–6.4]
	**34**	21 (0.46)	11.7 [6.5–21.2]	12.0 [6.6–21.7]	2.2 [1.1–4.0]
	**35**	28 (0.35)	10.0 [6.2–16.1]	10.1 [6.3–16.2]	2.1 [1.3–3.6]
	**36**	34 (0.21)	6.1 [4.0–9.3]	6.2 [4.1–9.5]	2.3 [1.5–3.6]
	**37**	34 (0.09)	2.4 [1.6–3.8]	2.5 [1.6–3.9]	1.8 [1.2–2.9]
	**38**	40 (0.04)	1.3 [0.8–1.9]	1.3 [0.8–1.9]	1.1 [0.7–1.7]
	**39**	48 (0.02)	0.9 [0.6–1.3]	0.9 [0.6–1.3]	0.9 [0.6–1.3]
	**40**	54 (0.03)	–	–	–
	**41**	39 (0.03)	1.1 [0.7–1.6]	1.1 [0.7–1.6]	1.1 [0.7–1.6]
	**42**	3 (0.05)	1.7 [0.5–5.4]	1.7 [0.5–5.5]	1.7 [0.5–5.5]
	**43**	1 (0.44)	12.3 [2.4–63.7]	12.6 [2.4–65.2]	12.0 [3.7–38.8]
	**Total**	**340 (0.48)**			
Post-neonatal mortality^d^	**32**	6 (0.35)	12.7 [5.5–29.7]	12.6 [5.4–29.3]	2.1 [0.9–4.9]
	**33**	15 (0.60)	21.5 [11.9–38.9]	21.1 [11.7–38.3]	3.25 [1.9–6.5]
	**34**	10 (0.22)	8.1 [4.3–15.3]	8.0 [4.2–15.2]	1.4 [0.7–2.6]
	**35**	7 (0.09)	3.2 [1.2–8.2]	3.2 [1.2–8.2]	0.7 [0.3–1.7]
	**36**	9 (0.06)	2.0 [1.0–4.1]	2.0 [1.0–4.1]	0.7 [0.4–1.5]
	**37**	30 (0.08)	2.8 [1.7–4.4]	2.8 [1.7–4.4]	1.8 [1.1–2.9]
	**38**	40 (0.04)	1.4 [0.9–2.1]	1.4 [0.9–2.1]	1.2 [0.8–1.8]
	**39**	60 (0.03)	1.1 [0.8–1.7]	1.1 [0.8–1.7]	1.1 [0.8–1.6]
	**40**	53 (0.03)	–	–	–
	**41**	27 (0.02)	0.8 [0.5–1.3]	0.8 [0.5–1.3]	0.8 [0.5–1.2]
	**42**	1 (0.02)	0.6 [0.1–4.7]	0.6 [0.1–4.7]	0.6 [0.1–4.4]
	**43**	0 (0.00)	NC	NC	NC
	**Total**	**258 (0.37)**			

*p* (Somer’s D) < 0.001: significant trend for mortality rates according to gestational age

^a^RR: relative risk

^b^aRR: relative risk adjusted for sex and pregnancy-related disease: ICD10 codes P01 and P02

^c^aRR_1_: relative risk adjusted for sex, pregnancy-related disease (ICD10 codes P01 and P02) and for hospitalization in the first 28 days of life

^d^Population of singleton infants alive at d29: *N* = 696 358

## Discussion

In France, 9.8% of live-born neonates with GA ≥ 32 weeks were admitted to hospital between d1 and d28. An inverse relationship between GA and hospital admission or admission in NICU remained up to 40 weeks. The results of this study, which are in keeping with other French and international publications [1–7, 12, 15, 22–25], also confirmed that there was an inverse relationship between GA and in-hospital death from 32 – 40 weeks of gestation. Interestingly, a recent English study suggested that poor outcomes among late and early PT were not only due to physiological immaturity but also to the biological determinants of preterm birth whether they are spontaneous or medically indicated [7].

Overall results of our study fit well with the recent ACOG committee statement which highlighted that implementation of a policy to decrease the rate of non-medically-indicated delivery before 39 weeks has been found to improve the neonatal outcome [10].

More specifically, the usual categorization of deliveries by GA partially erases the continuum of prognosis with GA, especially in the category of “term” infants (37–40 weeks) and it is not sufficient for individual clinical decisions and accurate public health information in moderate and early PT.

Post-date infants were more likely to require neonatal and post-neonatal hospitalization compared to infants born at 40 weeks and the 43 weeks group was also at increased risk of neonatal mortality while the 42 weeks group was not. Even if any conclusion about the 43 weeks group must take into consideration the very small sample size, this result suggests that continued efforts to prevent post-date births and their complications are needed [26].

The mean duration of stay in the different neonatal care units fell with increasing GA. However, given the large number of early PT concerned, the care provided to them in neonatal care units accounted for 51.5% of the 490 986 hospitalization days required by babies born after 31 weeks of gestation. This information is important for the optimal organization of perinatal health. Similarly, even though the rate of hospitalization among late PT is low, this population is large and any increase in the size of this population may significantly affect the need for hospital beds [27].

Moreover, the detrimental effect of birth before 38 weeks was not limited to the neonatal period since both the hospital admission rate and the in-hospital mortality rate were increased in the post-neonatal period (d28–d365) in our study. A recent study of birth certificates in California showed that the odds ratios (ORs) for any hospitalization within 365 days of life steadily declined with GA with the exception of 35 to 37 weeks of gestation for which ORs plateaued or even rose with increasing GA [11]. Another study [24] of infants born at ≥ 33 weeks of gestation similarly reported the greater risk of hospitalization among infants born at 36 weeks. In contrast, this French study showed a constant declining rate of hospitalization with GA in the first year of life including hospitalization during the neonatal period. As the rate of any hospitalization in the first year of life was greater in France than in California [11] it could be suggested that reducing hospital admissions and shortening the length of hospital stays in late PT may favor rehospitalization. This hypothesis deserves further studies.

Our study showed an excess mortality in the first year after delivery at 37–38 weeks. A Swedish cohort study [28], similarly showed excess mortality not only in the post-neonatal period but also in infants aged from 1 – 5 years and in young adults for births at 37–38 weeks. Even though the cause of post-neonatal deaths could not be ascertained in our study, it is worth noting that the main causes of post-neonatal hospitalization were infections (bronchiolitis, gastroenteritis, ear, nose and throat diseases) and that the risk of post-neonatal hospitalization was closely related to low GA. Overall, these results suggest that the reinforcement of preventive measures against infant infections should be applied not only in preterm infants but also in early PT.

The in-hospital mortality rate in our study (0.85/1000 live births) was only one fourth of the infantile mortality rate reported in Metropolitan France (3.28 per 1000 live births in 2011) by the French Vital Statistics obtained from death certificates [29]. The difference was greater in the first 28 days (0.48 vs 2.21/1000) than in the d29-d365 period (0.48 vs 1.07/1000). The main causes of death in the first year of life in France were perinatal diseases (48.7%) and congenital malformations or chromosomal anomalies (21.1%) in the period 2000–2008 [29]. Therefore, most of the discrepancies between the in-hospital death rate and the French infantile mortality rate were probably due to births not included in this study (congenital malformations/chromosomal abnormalities and preterm births before 32 weeks), which have a high mortality rate [30] as also to the exclusion of multiple births. Other causes of death outside the hospital area are sudden infant death syndrome (8.82% of infantile mortality in France), accidents or death from other causes [31, 32].

Our results showed that the risk of post-neonatal hospitalization gradually increased from 32 to 40 weeks of gestation, even after adjustment for sex, pregnancy-related disease and initial hospitalization. However, other characteristics associated with the risk of post-neonatal hospitalization could not be ascertained in our population and this represents a limitation of our study. In a selected population of late PT, Shapiro-Mendoza and colleagues [33] found that risk factors for subsequent readmission were NICU stay less than 4 days, breastfeeding, Asian/Pacific Islanders, firstborn infants, and public payers at the time of delivery.

Therefore, special studies are now needed to obtain more knowledge on the health status of preterm infants hospitalized in the first month of life in order to identify the most high risk infants for morbidity and readmission.

Another limitation of this study is the lack of data about insufficient prenatal care and deprivation, which were found to be independent variables associated with infant hospital attendance or in-hospital death during the first year of life in several studies [31, 32].

## Conclusion

This national study focused on more mature preterm infants and identified 40 weeks as the GA with the best prognosis in the first year of life. However, the universal value of 40 week as the optimal length of gestation cannot be inferred from this study as physiological factors may influence the physiological duration of gestation (multiplicity but also race, ethnicity) as well as medical practices. We therefore feel that other studies should be conducted in territories with different population characteristics and/or medical practices and/or perinatal health care systems.

The rates of hospital admission and in-hospital death were inversely related to GA below 39 weeks. This study confirmed that being born late preterm was a risk factor of increased morbidity and mortality but also extended this observation to infants born at early term. These findings should contribute to the redefinition of what constitutes term birth and to eventual modifications of medical practices. Non-medically indicated birth at 37 or 38 weeks should be discouraged [10] and conversely, the reason for medically indicated birth should be clearly indicated in medical files.
